# Account of the Medical School at Edinburgh

**Published:** 1801-06

**Authors:** 


### *To the Editors of the Medical and Phyfical Journal.*

GENTLEMEN,

AS the Medical School of Edinburgh has now attained a degree of celebrity fcarcely equalled, and certainly not furpaffed, by any fimilar feminary in Europe, fome account of thofe meritorious individuals, whofe genius and induftry have been fo fuccefsful in diffufing fo extenfively its well-earned fame, may not be regarded as unworthy of a place in your very refpectable Journal; I fhall therefore, if you pleafe, occafionally fend you fome biographical notices of fome of the Founders of the medical department of this Univerfity, with fome curfory remarks on their difcoveries and works.

The firft founder of the Medical School of this city was certainly the late excellent Dr. Alexander Monro, a man whofe great modefty, humanity, indefatigable induftry, and high profeffional talents, excited the love and admiration of his cotemporaries; and whofe works exhibit fuch profound refearches, important difcoveries, and great practical utility, as muft endear his memory to the prefent and to every future generation.

Dr. Alexander Monro was born in London, on the 8th of September, O. S. 1697. His father, Mr. John Monro, was

---

an eminent furgeon, and ferved in the army in that capacity. Soon after the birth of his fon he retired from the army, and fixed the fcene of his refidence in Edinburgh, where his profeffional fkill, his active induftry, and his conciliating deportment, foon eftablifhed him in extenfive practice. But although much occupied in the line of his profeffion, he devoted a great fhare of his attention to the education of his fon, whofe dawning genius he foon obferved, and with pleafure fuperintended. At this period Edinburgh afforded few opportunities for medical improvement, and Mr. Monro, who was well acquainted with this defect, and anxious to remove it, fondly hoped that that fpirit of diligence and inveftigation, which actuated his fon even from his infancy, if judicioufly directed, and furnifhed with the proper means of improvement, might in time capacitate him to impart that knowledge which was then fo great a defideratum.

Young Monro, diftinguifhed by active genius, and by great induftry, foon acquired every branch of literature at that time taught in the Univerfity of Edinburgh; and having early refolved upon the profeffion of medicine, we may fuppofe him to have been initiated in the preliminaries of that fcience, under the tuition of his father, a man well qualified to direct him, and deeply interefted in his fuccefs. Farther, however, in the career of improvement he could not then advance; for, at this period, no traces of a Medical School had exifted; there were indeed nominal profeffors, but there were neither ftudents nor public prelections. Young Monro, of courfe, found it neceffary to felect fome other field for profecuting his enquiries; accordingly, an extenfive plan of education, firft in London, afterwards in Paris and in Leyden, was judicioufly devifed and fuccefsfully carried into execution. During his refidence in thefe places, Monro's diligence in availing himfelf of every opportunity for improvement which his fituation offered, was indefatigable. To the moft eminent teachers of the times he repaired for inftruction; and among thofe whofe public prelections he attended, it will be fufficient to mention the names of a Chefelden, of a Hawkfby, Chomel, Bouquet, Thibaut, and the immortal Boerhaave. With Boerhaave he lived in habits of ftrict intimacy, and, on leaving Leyden, this truly great man amply attefted his profeffional fkill, and his penetrating genius.

Monro did not reft fatisfied with the knowledge derived from his attendance on thefe celebrated profeffors; eagerly defirous to excel in the profeffion which he embraced, he explored every collateral channel through which real knowledge could be obtained. He every where courted the intimacy of men confpicuous for profeffional fkill, or for literary attainments, ments, and he aſſociated with thoſe who had been proſecuting the ſame enquiries with himſelf. In a ſociety of this kind at London, he read an Eſſay on the Bones in general, which conſtituted the ground work of a future publication on that ſubject, a Treatiſe which alone is ſufficient to confer immortality on its author, and which, in point of practical utility and accuracy of deſcription, ſtands as yet unrivalled among works on Oſteology. Of his activity and ſkill as a practical anatomiſt, he at this time exhibited ſome elegant ſpecimens in preparations of different parts of the human body, which were preſented by his father to the Royal Colleges of Phyſicians and Surgeons, and ſo well received by them, that a Mr. Drummond, who was then nominal Profeſſor of Anatomy, aſſured him, that if the future progreſs of his ſon correſponded with theſe fruits of his induſtry, he would on his return to Scotland reſign the anatomical chair in his favour, and by devolving his charge upon ſo promiſing a ſucceſſor, convert his nominal dignity into an uſeful profeſſion.

Having in conſequence of Mr. Drummond's reſolution, the proſpect of ſoon filling the anatomical chair, there can be no doubt that this corner-ſtone of medical ſcience was paramount in Monro's mind to every other ſubject of enquiry; but to his cotemporaries his practice, and to us his writings, exhibit ſatisfactory proofs of his attention to every other branch of medicine, and while they maintain his title to the character of an accompliſhed anatomiſt, ſubſtantiate his claim to the reputation of an able phyſician.

Qualified in this manner for the duties of a practitioner, and for the office of a teacher, Dr. Monro returned to Edinburgh. In that place fame had reported his acquirements previous to his arrival, he was not of courſe permitted to remain long inactive. He had not reſided there many months, when, in the year 1720, he was called upon to give the firſt Courſe of Lectures on Anatomy and Surgery which was ever delivered in that city. For the execution of this arduous taſk he brought great zeal and conſummate talents, he could of courſe hardly fail in giving ample ſatisfaction; his ſucceſs indeed correſponded with the expectations of his warmeſt admirers. The accuracy of his demonſtrations, and the ingenuity of his phyſiological remarks, were equally conſpicuous; while the conſtant application of his ſubject to the practice of phyſic and ſurgery, rendered his prelections peculiarly valuable.

It is not detracting from the abilities of this eminent profeſſor to aſſert their inadequacy to diffuſe the fame of a ſchool which had to cope with ſo many rival ſeminaries of deſerved eminence, without ſome coadjutors to ſecond and to ſupport his exertions.

exertions. Senſible of this fact, his father, whoſe zeal for the eſtabliſhment of a Medical School here had acquired ſtrength proportionate to the probability of ſucceſs, prevailed on Dr. Alſton, the then King's Botaniſt for Scotland, to give a courſe of lectures on the Materia Medica. Dr. Alſton was a reſpectable aſſociate; but other branches of medicine ſtill remained to be illuſtrated. Monro, therefore, exerted his powers of perſuaſion to kindle in others that enthuſiaſm for enlarging the boundaries of medical ſcience with which he himſelf was animated. But a ſhort period elapſed when his endeavours in this reſpect were crowned with ſucceſs, and the young profeſſor ſoon found himſelf aſſociated with colleagues whoſe talents gratified his moſt ardent wiſhes; with a Rutherford, a Sinclair, a Plummer, and an Innes, names which will be long remembered, and are deſervedly conſpicuous in the annals of medicine.

Countenanced by the labours of theſe eminent men, and of their immediate ſucceſſors, Dr. Monro, with unremitting diligence, conſecrated his time and his talents to the improvement of medical ſcience for the period of half a century. During this long lapſe of years, he muſt have received the increaſing fame of a ſeminary of education with a delight which reſulted from a conſciouſneſs of its being, in a great meaſure, indebted to himſelf for its exiſtence; and before the termination of his diſtinguiſhed career, he found it inferior to none, and equalled by few, of the Medical Schools in Europe. Such was the conſpicuous reward of that aſpiring genius, who had given birth to the medical ſeminary, which before he withdrew from the public ſtage of life almoſt arrived at the acme of its celebrity.

We deem it not improper here briefly to ſketch the arrangement which he obſerved in his annual courſe of lectures on Anatomy and Surgery, which, with the greateſt aſſiduity, and without the leaſt interruption, he delivered to a crouded and an admiring audience for the period of forty years. This courſe laſted upwards of ſix months, and ſo great was the reputation he acquired as a teacher, that ſtudents flocked to him, not only from the moſt diſtant parts of the Britiſh dominions, but alſo from foreign nations.

He introduced his courſe with an hiſtorical ſketch of the progreſs of Anatomy from the earlieſt ages. In delivering this intereſting abſtract, the ſtrength of his memory, and his facility of expreſſion, were peculiarly conſpicuous. There are thoſe living who ſtill remember with what eaſe and fluency he gave a regular account of the moſt diſtinguiſhed Anatomiſts, from the earlieſt periods to the preſent times, mentioning the different improvements and diſcoveries, the exact periods at which which every difcovery was made, and the claims of different authors to the honours of particular difcoveries.

2. Next followed his lectures on Ofteology. After a luminous and full difcuffion of the ftructure, ufe, and difeafes of the bones in general, he entered upon the confideration of each in particular, demonftrating it to his pupils both fingly and in the fkeleton; and after fhewing its particular parts and ftructure, he treated of its ufes, of the difeafes and accidents to which it is fubject.

3. He demonftrated all the mufcles of an adult fubject, with the vifcera of all the different cavities of the human body, and fhewed the nerves and blood-veffels in the bodies of children. After demonftrating each organ, he always treated of its phyfiology and pathology, illuftrating the difeafes to which it is liable, and enumerating the moft approved remedies. In this divifion of his courfe he alfo exhibited preparations of the different parts, as he treated of them.

4. After finifhing the anatomical demonftrations of the human body, he endeavoured to illuftrate ftill farther this interefting fubject, and to throw fome more light on the animal œconomy, by the diffection of different animals, quadrupeds, fowls, and fifhes, and by comparing the ftructure and ufe of their organs, with the correfponding organs in man.

5. He confidered particularly the difeafes for the removal of which chirurgical operations are commonly neceffary, and the beft methods of treating them. He then fhewed to his pupils the different operations of furgery performed on the dead fubject, and mentioned the various methods which had been propofed for performing thefe operations, with the advantages and difadvantages attending each.

6. After the operations of furgery, he fhewed the different bandages and machines ufed by furgeons, with the mode of their application, and mentioned the cafes in which they were ufeful.

7. He clofed his long and laborious winter courfe with fome general lectures on the phyfiology of the human frame.

We have thus briefly adverted to the plan which the celebrated Monro adopted in his Lectures, becaufe it exhibits a confpicuous proof of his great judgment, of his extenfive and various information; and becaufe it may fafely be propofed as a model for the imitation of other anatomical demonftrators.

Hitherto we have contemplated this eminent man, chiefly in his profeffional capacity; but to regard him in this light alone would be but exhibiting a partial view of his character and conduct. In the labours connected with his department in the Univerfity, his diligence and anxiety to excel were indeed exemplary; emplary; but he was no lefs affiduous in adding to the ftock of his own knowledge, and in the improvement of medicine in its various branches, both as a fcience and as an art. He had long and ferioufly reflected on the manifold advantages which would accrue to ftudents in medicine, to the country at large, and indeed to fociety in general, from the eftablifhment of an hofpital in the city of Edinburgh. To impart immediate relief to the unfortunate whom the accumulated load of difeafe and of poverty crufh to the ground; to illuftrate the healing art both by experiment and by example; to impart ufeful inftruction not only to the ftudent but alfo to the practitioner, and even to remove fome of thofe difficulties which impede the progrefs of medicine itfelf, were the invaluable confequences in which it was reafonably expected fuch an eftablifhment would refult.

The mind of this benevolent man was impreffed with this view of the fubject; and in order to accomplifh the great work, he had recourfe to every expedient which an active genius, moved by compaffion for the miferies of his race, and interefted in every feafible plan for their mitigation, could fuggeft. He wrote a pamphlet relative to the advantages which would accrue to the community from fuch an inftitution, and the impreffion it produced on the public was fuch as to intereft every denomination of people in the undertaking, from the firmeft conviction of its being calculated, not only to accommodate the poor and the needy, but to advance the public good. Thus was the Hofpital erected by the joint co-operation, not only of thofe whom heaven had bleffed with enlarged views and with feeling hearts, but even of others, whom the partiality of fortune had placed in affluent circumftances, and whom, although but flightly impreffed with the defire of alleviating the miferies of their poor brethren, the perufal of Dr. Monro's pamphlet had convinced of the utility of fuch an eftablifhment.

The limits of this paper will not allow us to defcant upon the character of any other individual, who gave an active countenance to Monro, in realifing this great national charity, elfe honourable mention might and indeed ought to be made of many benevolent characters, and more efpecially of the late George Drummond, Efq. who had often occupied the chair of chief magiftrate of the city of Edinburgh, with no lefs credit to himfelf than advantage to the public, and for whofe liberal principles, and inceffant endeavours for the eftablifhment of the Royal Infirmary, his memory will be long held in facred remembrance by a grateful pofterity. The tafk of defigning, fuperintending, and executing every part of this great work, was devolved by the firft contributors upon this active magiftrate, giſtrate and upon Dr. Monro, under the deſignation of the building committee; and by the joint exertions of theſe two meritorious individuals, an hoſpital, large, elegant, and commodious, was ſoon provided with every accommodation for its poor, diſeaſed, and deſtitute inmates. To their mutual labours therefore this country is indebted for all the benefits derived from the Royal Infirmary of Edinburgh.

In theſe active exertions Dr. Monro looked forward to the many benevolent purpoſes which a public hoſpital would ſubſerve, but his attention was chiefly engroſſed by the advantages which would accrue from it as a field for medical education; and to the attainment of this great object, he devoted an active ſhare of his labours to the lateſt period of his life. When burdened with thoſe infirmities which labour and age had faſtened upon him, he retired from his claſs, and conſigned to his ſon the charge of the Anatomical Theatre, he was ſtill aſſiduous in his attendance in the Hoſpital, and continued to give Clinical lectures, with great ſkill and with indefatigable induſtry. In the treatment of his patients his practice was rational; and his remarks on the caſes which came under his view, indicated the acuteneſs of his intellect, and the ſolidity of his judgment. Even when baffled in the efforts of his medical ſkill, diſſection furniſhed him with an opportunity of imparting many uſeful leſſons to his pupils. The knowledge of the cauſes of diſeaſe, by diſſection, was an object which occupied much of his attention, and in the inveſtigation of which he embraced every opportunity which his different ſituations as phyſician, lecturer, and manager of the hoſpital afforded him. At the inſpection of his dead patients he was always preſent; and not only dictated an accurate report of the diſſection, but, with a nice diſcrimination, contraſted the diſeaſed and the ſound ſtate of the organ.

From his official capacity as an Anatomical Demonſtrator, and a Clinical Profeſſor, he had many opportunities for experiments, both on the living and on the dead ſubject; from theſe opportunities he was eminently calculated to derive every poſſible advantage, and the fruits of his labours he conſecrated to the improvement of his favourite ſcience.

We have thus delineated Dr. Monro's character as a Profeſſor, a Phyſician, and as a Founder of our National Hoſpital. That ardent and upright temper of mind which diſtinguiſhed him in theſe public functions, marked his conduct in every other department of life. His practice as a medical man was very extenſive. He was a member of many learned ſocieties, and ſuperintended the management of many public charities. Theſe engagements, however extenſive and multifarious, did

 not not preclude him from attending to other public concerns, both of a civil and of a political nature. In ſhort, the purſuits in which this great man was engaged, perhaps exceeded, in point of variety and importance, the avocations of any of his cotemporaries; yet did he diſcharge the duties relative to them all, with the ſtricteſt integrity and with the moſt rigid punctuality.

This eminent perſon was at laſt attacked by a moſt painful and lingering diſtemper, the torments of which he endured with a pious reſignation and with a ſteady fortitude. To this mortal diſeaſe, whoſe progreſs could not be arreſted by the utmoſt efforts of medical ſkill, or of human aſſiſtance, after many months ſufferings he fell a victim, and cloſed his moſt active and uſeful career on the 10th of July, 1767, in the 70th year of his age. — We ſhall now cloſe this ſuperficial ſketch of his life with a brief notice of his diſcoveries and works.

A circumſtantial review of this learned Profeſſor's numerous diſcoveries and valuable improvements, both in the ſcientific and practical departments of the healing art, would lead us longer into detail than is conſiſtent with this brief memorial. We have ſeen in what degree of eſtimation he was held by his cotemporaries, and poſterity will recognize his ſuperlative merits in his writings, when every biographical notice regarding him ſhall have periſhed in the ſtream of oblivion. Of every ſociety in Edinburgh, inſtituted either for the improvement of the arts, or for the diffuſion of the ſciences, he was juſtly regarded one of its ableſt ſupporters, and of its brighteſt ornaments. He was a member of the Colleges of Surgeons and Phyſicians; of the Medical and Philoſophical Societies; of a ſelect Society conſtituted for the purpoſe of diſcuſſing moral and political queſtions, and of the Society for promoting Arts, Sciences, and Manufactures in Scotland. In the diſcuſſions of all theſe various bodies he engaged with an active and an uniform ardour, and his zeal correſponded with his pre-eminent talents. Thus deſervedly eſteemed and reſpected at home, he was equally revered and honoured abroad. Of the Royal Society of London he was a non-reſident member; and the Royal College of Phyſicians at Paris enrolled his name in the catalogue of its foreign aſſociates. — Let us now take a curſory view of his writings.

His Treatiſe on Oſteology was originally deſigned to facilitate the progreſs of ſtudents in this fundamental branch of anatomical knowledge, but it merits attention from the greateſt adept in the ſcience; and from its peruſal the moſt experienced practitioner may derive uſeful information. Every perſon who is acquainted with this invaluable treatiſe, muſt indeed regard it as a monument of its author's abilities, exhibiting at once once the moft luminous proofs of extenfive reading, accurate information, and judicious reflection. The defcription of the bones is minute, exact, and accordant with nature; the fentiments of other authors are faithfully narrated, and candidly appreciated; and the work every where abounds with new and important obfervations, which have an immediate reference to practice. This great work met with the deferved reception.—Eight large impreffions were fold during the author's life-time. It has been tranflated into moft European languages; and the French edition, in folio, publifhed by the celebrated Monfieur Sue, Demonftrator to the Royal Academy of Sculpture and Painting at Paris, is one of the moft fuperb books in print, and adorned with as elegant and mafterly engravings as are to be found in any anatomical work.

To the latter editions of his Treatife on the Bones, he added a Neurology, or Anatomy of the Nerves, in which he gives a concife and accurate defcription of the larger branches of thefe conveyers of fenfe and motion. As this work was alfo written for the improvement of his pupils, he has not delineated the minuteft branches, being afraid that his going into fuch particular details might embarrafs the minds of the young enquirers, rather than impart inftruction. In this treatife he has alfo mentioned moft of the prevailing opinions concerning their ftructure and ufe; and although fucceeding anatomifts have called in queftion his fpeculations relative to the texture and phyfiology of thefe important organs, it muft be confeffed that the arguments adduced in fupport of his opinions are equally fpecious and ingenious. To this treatife he has alfo fubjoined an account of the receptacle of the chyle, and of the thoracic duct, organs of effential importance in the animal œconomy, which, in point of accuracy, has not been furpaffed by any fucceeding author.

We are now to confider him as a contributor to, and the editor of, another and more extenfive medical work. In the year 1731, the profeffors of medicine, and other phyficians and furgeons in the city of Edinburgh, conftituted themfelves into a fociety for collecting and publifhing fuch medical obfervations and effays as the members themfelves could bring forward, or as might be communicated to them by friends and correfpondents. To this fociety Dr. Monro acted in the capacity of fecretary. At the beginning of the inftitution the members punctually attended, and remarked upon the papers fubmitted to their infpection: but after the publication of the firft volume, they grew remifs in the difcharge of their duty, and not long thereafter, the whole labour both of collecting and publifhing their tranfactions was devolved upon the fecretary. Of the papers of this collection

Yyy 2 Doctor

Doctor Monro furniſhed many more than his ſtrict quota; and in theſe excellent eſſays he has added materially to our knowledge, both of the ſtructure and phyſiology of ſeveral parts of the human body. His anatomical knowledge ſuggeſted to him many uſeful and practical inferences, and he propoſed many new improvements in the mode of performing many capital operations, the greater part of which have been adopted by our moſt eminent ſurgeons. To Doctor Monro, therefore, are we chiefly indebted for the ſix volumes of Medical Eſſays and Obſervations, a work which has been ſingularly approved of by the moſt competent judges, undergone various editions in the Engliſh language, and has, moreover, been tranſlated into many foreign languages. The very univerſal reception with which this work has been deſervedly honoured, ſuperſedes the neceſſity of our approbation: ſuffice it to notice, that honourable mention is made of it by the immortal Haller, who appreciated its intrinſic merit ſo highly as to declare, that it ought to find a place in the library of every medical practitioner *; and indeed it muſt be confeſſed, that it has enriched every department of medical ſcience with numerous and important diſcoveries. The plan of this ſociety was afterwards enlarged by the admiſſion of ſeveral other gentlemen, eminent for literary and philoſophical talents, and by this arrangement, its tranſactions became philoſophical as well as medical. When this ſociety was thus new modelled, Dr. Monro was elected one of its preſidents. Their papers were publiſhed under the deſignation of Literary and Phyſical Eſſays; and in the two firſt volumes are to be found ſeveral papers from the Doctor's pen, which indicate the ſtill exuberant treaſures of his mind, and adorn the pages of that valuable collection.

His account of the ſucceſs of inoculation in Scotland cloſed his career as an author. This paper was originally written in anſwer to an application from the delegates, upon whom the faculty of phyſicians at Paris had devolved the taſk of appreciating the merits of this practice. It exhibits a ſtriking proof of his extenſive correſpondence, and of his indefatigable induſtry. It had a conſiderable effect in removing prejudices, which at that period were general and ſtubborn, and in reconciling both practitioners and parents to a ſimple ſalutary operation, that has been the means of preſerving the lives of thouſands, by diſarming, in a great meaſure, a malignant diſtemper of its deadly virulence.

* Quinque volumina Speciminum Societatis Edinburgenſis prodierunt (quorum ultimum duplex eſt) Medicis perutilia, et Chirurgis, et Anatomicis. Monrous ibi eminet. Haller, Meth. Stud. Med. p. 69.

Some

Some of his poſthumous works which have appeared are diſtinguiſhed by that elegance and ſimplicity of expreſſion, and by that extent of information which characterize his other maſterly productions. In his Oratio de Cuticula Humana, many curious circumſtances are deſcribed, which eſcaped the obſervation of former anatomiſts, particularly the appearance of the fibres which connect the cuticula with the *cutis vera.* In the Eſſay on Comparative Anatomy, originally compoſed from notes taken down from his lectures, but found in a more correct form among his papers, and publiſhed ſince his death in the quarto edition of his work, he certainly evinces himſelf a great maſter. It cannot, indeed, be regarded as a complete work; but the plan is judicious, and the proper tract for inquiry pointed out, which might be eaſily proſecuted. The deſcriptions are taken from the life, and the reaſoning is intereſting, perſpicuous, and concluſive. As in the other works of the author, ſo in this treatiſe we find many practical obſervations on ſurgery and medicine interſperſed, which muſt equally inſtruct and entertain the reader.

Beſides theſe two poſthumous publications, it is underſtood he left ſeveral other manuſcripts, which have not as yet ſeen the light. We have heard of a Hiſtory of Anatomical Writers.—An Encheireſis Anatomica—Heads of Lectures—A Treatiſe on Wounds and Tumours—Obſervations on ſome parts of Heiſter's Surgery. Theſe papers have not been inſerted in the poſthumous edition of his works, although there can be little doubt of their poſſeſſing intrinſic merit; and the deſerved celebrity of the author would have certainly ſecured them a favourable reception.

We have thus briefly reviewed the life and writings of the late Doctor Alexander Monro, and it may, perhaps, be expected from us to cloſe the narrative with a delineation of the prominent features of his character. The lineaments of his mind have, in ſome meaſure, been traced in the courſe of our narration, and to exhibit the aggregate of his qualifications within the limits which muſt be preſcribed to this eſſay, would be impoſſible; we ſhall therefore reſt ſatisfied with obſerving, what indeed has already appeared in his hiſtory,—that the zeal and induſtry diſplayed by him in the proſecution of knowledge have ſeldom been equalled, perhaps never ſurpaſſed; that he taught his favourite ſcience with an enthuſiaſm and a liberality of ſentiment proportioned to its vaſt importance; that he was conſpicuous for active philanthropy and mildneſs of temper; that he was ſteady in his friendſhips and frank in his intercourſe, a dutiful ſon, an affectionate father, and an excellent huſband; that he was always forward to patronize modeſt neglected merit, and to relieve the exigencies of indigent genius. It is not contended, that in the line line of his profeffion Doctor Monro has fuperfeded the neceffity of future induftry, but it is maintained that he has accomplifhed more than the fhort fpan of human life could well authorize us to expect from the exertions of any fingle individual; and that in the various ftations of a Student, a Teacher, and a Practitioner in Medicine, he has exhibited a bright pattern for the imitation of pofterity.

*Edinburgh, 9th April, 1801.* D. F.

---

### *To the Editors of the Medical and Phyfical Journal.*

GENTLEMEN,

